# Chronic Ectopic Pregnancy Mimicking Broad Ligament Fibroid: A Case Report

**DOI:** 10.7759/cureus.72245

**Published:** 2024-10-23

**Authors:** Yashika Jaiswal, Anuja Bhalerao

**Affiliations:** 1 Department of Obstetrics and Gynaecology, NKP Salve Institute of Medical Sciences and Research Centre, Nagpur, IND

**Keywords:** broad ligament fibroid, chronic ectopic pregnancy, differential diagnosis, exploratory laparotomy, pelvic tumour

## Abstract

Chronic ectopic pregnancy (CEP) is the implantation of the conceptus outside of the uterine cavity and fallopian tubes are the most frequent site. CEP is among the leading causes of maternal mortality and morbidity. The diagnosis is sometimes made at a later gestational age and is often missed pre-operatively and made intra-operatively, which may cause organ damage and significant hemorrhage. Hence, the present report highlighted a rare case of CEP in a 28-year-old woman who was married for six years and presented to the Department of Obstetrics and Gynaecology with the chief complaints of bleeding per vaginum since 15 days, soaking three to four pads per day. The menstrual history reported the presence of intermenstrual bleeding and was not associated with the passage of clots along with no preceding amenorrhea. The urine pregnancy test (UPT) was weakly positive. For diagnostic assessment, in the view of weak positive UPT describing the possibility of CEP and MRI which was suggestive of broad ligament fibroid. The exploratory laparotomy was planned to confirm the diagnosis and as a part of the intervention which confirmed the diagnosis of CEP mimicking broad ligament fibroid. In conclusion, it is essential to consider CEP in the differential diagnosis of pelvic tumors. For such cases, a combination of clinical vigilance, appropriate imaging, and surgical intervention is essential for effective management.

## Introduction

The conceptus when implanted outside of its typical location within the uterine cavity is known as a chronic ectopic pregnancy (CEP) and fallopian tubes are the most frequent site [1]. It affects 2% of pregnancies overall, and 95% of cases occur in the fallopian tube [2]. The cervix, ovary, abdomen, and scar from a cesarean section are the other sites of implantation [3,4]. CEP can be acute or chronic and the most common symptoms involve abdominal pain in the lower quadrant, amenorrhea, and vaginal bleeding [5,6]. However, CEP, characterized by progressive tubal wall disintegration, is not well-defined and has a rare occurrence [1].

A massive, unruptured mass consisting of several minor hemorrhages in the cavity of the peritoneum along with the development of a huge complicated pelvic mass can be observed in a CEP. Diagnosis of CEP may mimic endometriosis, vascular tumors, acute pelvic inflammatory disorders, and pelvic abscess [7]. CEP is among the primary causes of maternal mortality and morbidity. The diagnosis is frequently derived during surgery, occasionally at a later age of gestation, as it is often missed preoperatively, which increases the risk of serious organ damage and hemorrhage [8,9]. Death, shock, and intraperitoneal hemorrhage may result from a missed diagnosis [8]. Hence, the present report describes a rare case of CEP mimicking broad ligament fibroid.

## Case presentation

Patient information

A 28-year-old woman married for six years, with parity two live two, all full-term normal delivery presented to the Department of Obstetrics and Gynaecology with the chief complaints of bleeding per vaginum since 15 days, soaking three to four pads per day. The menstrual history reported the presence of intermenstrual bleeding and was not associated with the passage of clots along with no preceding amenorrhea. The urine pregnancy test (UPT) was weakly positive.

Clinical examination

The vital signs were steady upon assessment. Per abdomen examination reported that the abdomen was non-tender, soft, and had no palpable organs/masses. Per speculum examination revealed no abnormalities in the vagina and cervix. An attached mass to the uterus of 9-10 cm was observed in per vaginal examination. Although there was fullness in the Douglas pouch, there was no pain.

Diagnostic assessment

**Figure 1:**
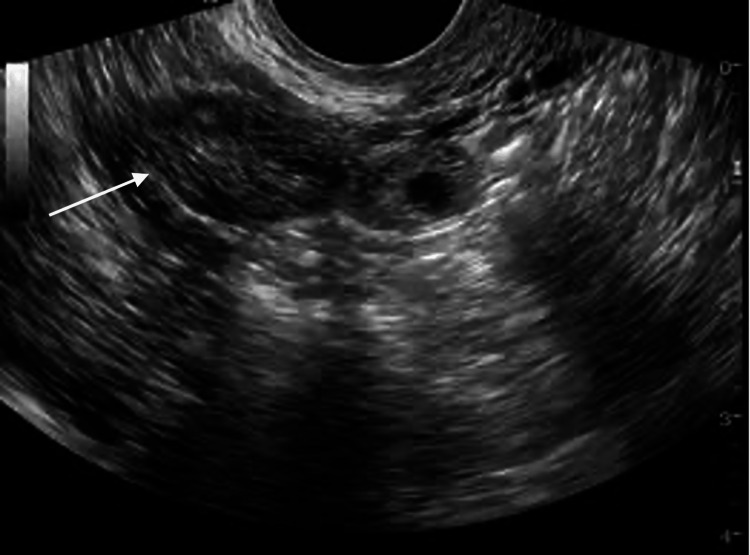
An arrow pointing to an ill-defined heterogenous large lobulated lesion measuring 9 x 4.9 x 2 centimeters in the ultrasonography of pelvis

Ultrasonography (USG) of the pelvis showed an endometrial thickness of 1.6 cm, which was thickened and heterogenous in nature. The USG report described an ill-defined heterogenous large lobulated lesion measuring 9 x 4.9 x 2 cm in the left adnexa in close proximity to the anterior myometrium of the uterus which concluded the possibility of broad ligament fibroid or subserosal fibroid as shown in Figure 1. Additionally, pelvic magnetic resonance imaging (MRI) showed free fluid level in the intrauterine sac demonstrating the possibility of broad ligament fibroid. Serial beta-human chorionic gonadotropin (bHCG) was performed which was 175 IU/ml on admission and after two days it was 179 IU/ml. Because of weak positive UPT describing the possibility of CEP and MRI which was suggestive of broad ligament fibroid, exploratory laparotomy was planned to confirm the diagnosis and as a part of the intervention.

Therapeutic intervention

**Figure 2:**
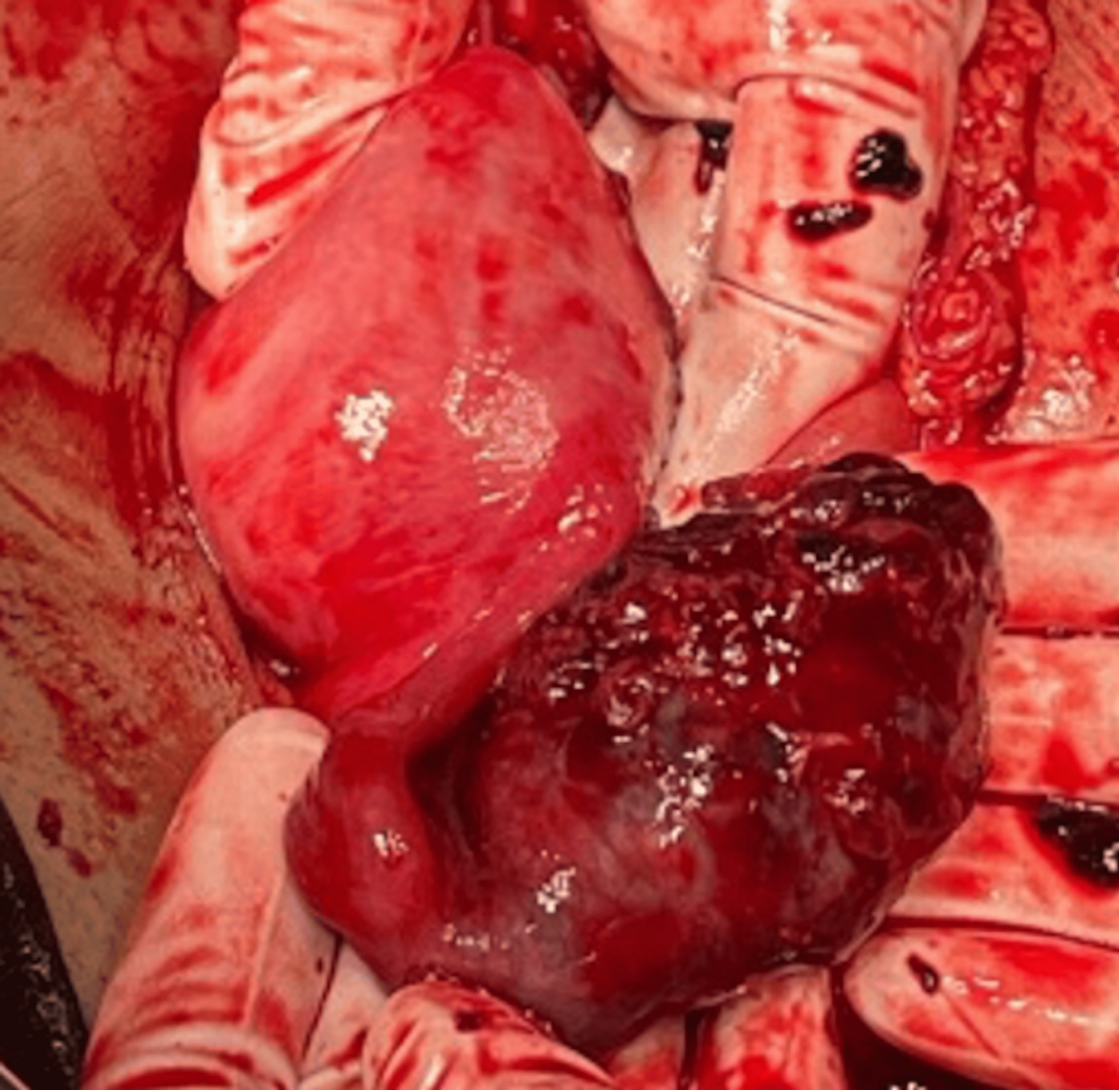
The left fallopian tube measuring 6 x 4 x 3 centimeters

On exploratory laparotomy, hemoperitonium was present. Organised clot of 160 gm was removed from pouch of Douglas. Left fallopian tube measured 6 x 4 x 3 cm as shown in Figure 2. CEP was present for which suction and evacuation was performed. The left fallopian tube was found to be dilated and contained blood in gross pathology. There were two distinct primary defects found in the tube wall. Microscopic analysis revealed immature chorionic villi, which revealed the ectopic pregnancy.

## Discussion

The present case demonstrated the diagnostic dilemma associated with both broad ligament fibroid and CEP since the patient had a weakly positive UPT and an extensive pelvic mass. Diagnosis of chronic ectopic requires experience. UPT is the most cost-effective and first-line investigation to rule out ectopic pregnancy. On the other hand, a low serum HCG level results from the small amount of viable chorionic villi in a CEP. Because of this, the UPT might remain negative in these circumstances, mistakenly redirecting away from the possibility of a CEP diagnosis [1]. Serum beta-human chorionic gonadotropin is usually in the low ranges, due to the destruction of viable trophoblastic tissue as observed in the present case. Therefore, it is essential to diagnose CEP as soon as possible to avoid the emergence of additional adhesions and to put together for corrective surgery [10].

Azhar et al. described a case report on ruptured broad ligament ectopic pregnancy where a 27-year-old woman presented to the emergency department with severe right lower quadrant pain and amenorrhea with UPT positive. Differential diagnoses were ruptured ectopic pregnancy, and degenerating fibroids. Active bleeding necessitated an open laparotomy following a diagnostic laparoscopy. After the adhering tissue was removed using vascular implants from the right broad ligament and the pelvic arteries, pathology was notified. Microscopic examination revealed rare immature chorionic villi in the broad ligament, compatible with confirmation of ruptured ectopic pregnancy [8]. In a study conducted by Singh, 12/15 (80%) women had a presenting complaint of either abnormal uterine bleeding (AUB) or abdominal pain, or both. The UPT was found to be positive in 11 patients. An imaging study reported that CEP appears as a heterogeneous mass in the pouch of Douglas or adnexa for which differential diagnosis of pelvic inflammatory disease (PID), endometriosis, and fibroid uterus should be considered [1].

CEP has a gynecological masquerade as it is often misdiagnosed preoperatively. A range of interventions are available, such as hysteroscopy, laparoscopy, and laparotomy; a variety of image-guided procedures are developing; and a growing number of pharmaceutical drugs are available. Each of these interventions has an application for a patient who was selectively selected and counseled [11,12]. In the present case, exploratory laparotomy allowed for accurate diagnosis and effective management with minimal morbidity. In summary, for a young patient with AUB, low b-HCG, weak positive UPT, and no history of preceding amenorrhea, the diagnosis of CEP should be considered.

## Conclusions

The present case report highlighted a rare case of CEP mimicking broad ligament fibroid that led to a diagnostic dilemma. Additionally, the case illustrated the importance of considering CEP in the differential diagnosis of pelvic masses. For such cases, a combination of clinical vigilance, appropriate imaging, and surgical intervention is essential for effective management. Hence, enhanced awareness and reporting of rare presentations similar to the present case would contribute to better diagnostic strategies and patient outcomes in the future.
